# Distinct strategies of soil bacterial generalists and specialists in temperate deciduous broad-leaved forests

**DOI:** 10.1128/aem.00992-25

**Published:** 2025-07-30

**Authors:** Xueying Li, Haixia Li, Senlin Wang, Huiping Zhang, Yizhen Shao, Yun Chen, Zhiliang Yuan

**Affiliations:** 1 College of Life Science, Henan Agricultural University 70573, Zhengzhou, China; 2 Baotianman National Nature Reserve of Hennan, Nanyang, China; University of Delaware, Lewes, Delaware, USA

**Keywords:** soil bacteria, niche breadth, habitat generalists, habitat specialists, community assembly process, co-occurrence network

## Abstract

Based on global biotic homogenization, habitat generalists and specialists play an important role in maintaining the stability of ecosystems. However, limited information is available about the assembly processes and co-occurrence patterns of soil bacterial habitat specialists and generalists in forest ecosystems, particularly their response mechanisms to environmental factors. In this study, high-throughput sequencing technology was used to investigate the role of the ecological assemblage processes of soil bacterial habitat specialists and generalists and their role in maintaining the stability of the symbiotic network in temperate deciduous broad-leaved forests (China). The results showed that compared with specialists, the diversity of bacterial habitat generalists was lower, but their distribution ranges and environmental niche breadth were wider. Results from the null and neutral models indicate that, compared to deterministic processes, the community assembly of habitat generalists and specialists is more strongly influenced by stochastic processes, with generalists exhibiting a higher degree of stochasticity than specialists. Network analysis results showed that habitat specialists played a greater role in maintaining the stability of the bacterial co-occurrence network than the generalists. In addition, bacterial habitat specialists were more likely to be affected by light and spatial feature vectors than generalists. These findings provide a novel perspective for understanding the assembly processes and diversity maintenance mechanisms of the forest soil bacterial community.

**IMPORTANCE:**

Limited information is available about bacterial specialists and generalists in forests. Generalists were more affected by stochastic processes than specialists. Specialists played a more important role in network stability than generalists. Light and spatial vectors had stronger effects on specialists than generalists.

## INTRODUCTION

In natural ecosystems, most studies have shown that microbial communities are classified as habitat generalists, specialists, and other taxa depending on their niche breadth (1, 2). Habitat specialists with narrow niche breadth are considered more competitive but less resistant against changing environments, while the habitat range of generalists and their fitness for a particular environment are more extensive (3, 4). In comparison with generalists, habitat specialists have a faster worldwide decline based on niche breadth predictions, contributing to the functional homogenization in biodiversity (5). This homogenization might change ecosystem functioning, thus endangering ecosystem services (6). In addition, some generalists exhibit a rapid rate of niche evolution (7, 8). To some extent, the change of niche breadth can reflect extinction risk (6). Based on global biotic homogenization, habitat generalists and specialists play an important role in maintaining the stability of ecosystems (5).

Ecologists have extensively studied the environmental factors controlling soil microorganism abundance and distribution patterns (9, 10). Results showed that pH, temperature, and salinity are the key environmental factors that control the compositions and distributions of microbial habitat generalists and specialists (11). In addition, light has been shown to significantly influence soil microbial biomass and alter the structure of soil bacterial communities (12). Habitat specialists and generalists of aquatic invertebrates have different ecological responses to environmental changes in various ecosystems. Habitat specialists respond with greater variance to environmental conditions, while generalists respond with less variance to environmental changes (13). In aquatic ecosystems, environmental factors such as salinity, temperature, and total nitrogen have a greater effect on the habitat specialists of microbial communities because specialists might have strict requirements for environmental conditions (11, 14). Habitat specialists may face extinction if drastic environmental disturbances occur (15). However, studies have not established how habitat generalists and specialists of soil bacterial communities differ in response to environmental changes, particularly in forest ecosystems.

Soil bacteria are important drivers of forest biogeochemical processes and play an important role in regulating nutrient cycling and promoting plant growth (16). Bacterial diversity is strongly associated with multiple ecosystem functions, such as climate, habitat disturbance, vegetation, and soil properties of environmental changes (5). The study of these changes is essential for the development of basic ecological theory and predicting the response of ecosystems to environmental change (17, 18). Bacterial diversity in forest ecosystems has received increasing attention (19).

The understanding of the ecological processes of bacterial community aggregation is a continuing subject of debate in the microbial ecology field (14). Bacterial community assembly can be divided into deterministic and stochastic processes (20). Deterministic processes involve environmental filtering (e.g., salinity, pH, and temperature) and biotic interactions (e.g., competition, predation, mutualism, and trade-off) (21, 22). By contrast, stochastic processes include dispersal limitation and random changes (e.g., birth, death, ecological drift, extinction, and speciation) (23). Habitat generalists and specialists of microbial communities in lake sediments in Tibetan lakes are mainly affected by stochastic processes (11). Stochastic processes determine the assembly of micro-eukaryotic community habitat generalists in an anthropogenically impacted river, while the deterministic processes strongly influence the distribution of habitat specialists (14). These inconsistent results suggest differences in community assembly between habitat specialists and generalists that have been attributed to differences in ecosystem type (24, 25). In addition, environmental factors can regulate the balance between deterministic and stochastic processes (26). For example, soil pH and soil moisture content are major drivers that regulate the balance between deterministic and stochastic processes of abundant and rare bacterial subcommunities, respectively (27). Low salinity contributes to the dominance of stochastic processes in micro-eukaryotic plankton community assembly (28). However, researchers have not determined the key environmental factors that regulate the balance between habitat generalists and specialists in community assembly mechanisms in soil bacterial communities of temperate deciduous broad-leaved forests.

The complex interspecific interactions within microorganisms are extremely important for maintaining microbial diversity and ecosystem function (29, 30). At present, most studies have used co-occurrence networks to explore the structure of complex microbial communities and interactions between microorganisms (30), such as oil-contaminated soils (31), rivers (14, 32), and marine water (33). Co-occurrence networks can be used to clarify interactions between microbial taxa, identify keystone species, compute network topological features, and further provide useful information for exploring species coexistence and microbial diversity (11). Additionally, microbial communities can be divided into modules comprising highly interconnected microorganisms, with modularity interpreted as habitat heterogeneity, niche overlap, and phylogenetic correlation (34). However, the co-occurrence patterns of generalists and specialists in temperate forest soil bacterial communities have not been fully understood.

In the present study, 16S rRNA gene amplicon sequencing was used to sequence bacterial communities from 120 soil samples collected from the forest dynamic monitoring plots of Baiyunshan. The park is rich in species resources and generally belongs to well-preserved natural ecosystems. Baiyunshan National Forest Park (Henan Province, China) is a typical forest ecological zone in the transition zone between the warm temperate and subtropical zones because of its unique geographic location and complex topography, providing an ideal location to study the distribution pattern and ecological process of bacterial communities (18). The distribution pattern of soil bacterial communities has aroused great concern among ecologists (18, 35), but the distribution pattern and mechanism of soil bacterial subcommunities with different niche breadth on temperate forest plots remain poorly understood. The objectives of this study are as follows: (i) to reveal the community assembly mechanisms of soil bacterial community habitat generalists and specialists and analyze the relative importance of stochastic and deterministic processes; (ii) to explore the coexistence patterns of the habitat generalists and specialists; (iii) and to assess the effects of light, plant, topography, and spatial eigenvectors on habitat generalists and specialists. We hypothesized that stochastic processes play a greater role in the community assembly of habitat generalists than specialists. Habitat specialists contribute more to network stability than generalists. In addition, considering the low environmental tolerance of habitat specialists, habitat specialists might be more susceptible to environmental factors than generalists.

## MATERIALS AND METHODS

### Study site

The sampling site is located in Baiyun Mountain National Forest Park (111°47'–111°51'E, 33°38'–33°42'N) in Henan Province, China. The park has an area of approximately 168 km^2^ at elevations from 800 to 2,216 m above sea level and is located in the transition region from warm temperate zone to north subtropical zone (36, 37). Its annual mean temperature is 12.2°C, the extreme maximum temperature is 41.2°C, and the extreme minimum temperature is –14.4°C. The annual mean rainfall is 1,200 mm, mostly from July to September. Baiyun Mountain National Forest Park is rich in plant resources, with an average forest coverage of 81.2% and approximately 1,991 plant species (38). In the present study, *Quercus aliena* var. acuteserrata, *Toxicodendron vernicifluum*, and *Sorbus alnifolia* are some dominant tree species in temperate deciduous broad-leaved forests.

### Sampling point setting and sample collection

**Fig 1:**
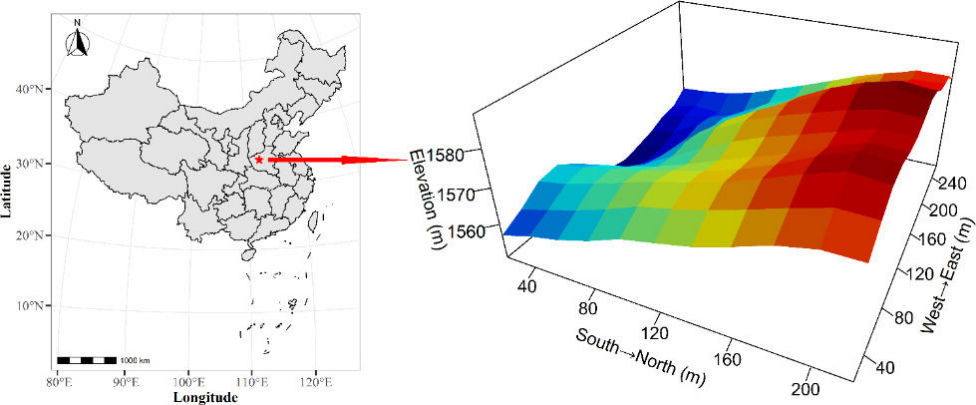
Location and topography of the 4.8 hm^2^ forest dynamic plot in Baiyun Mountain National Forest Park. The map was created using ArcGIS, with the basemap sourced from the official China Standard Map Service, under map review number GS(2019)1822.

According to the construction standards of the Smithsonian Institution’s Center for Tropical Forestry Research (39), a long-term fixed monitoring plot of 4.8 hm^2^ with a length of 240 m from east to west and 200 m from north to south was established in the Baiyunshan National Forest Park. The 4.8 hectare plot was divided into 120 quadrats (400 m^2^ each, Fig. 1). Three soil sub-samples were collected from each 20 m × 20 m square (10 m distance among the three sub-samples), and then the three soil samples were mixed evenly into one soil sample. A total of 120 soil samples were collected in this sampling campaign. Each soil sample was divided into two parts; one was used for soil chemical analysis, and the other was stored at –80°C for bacterial microbiological analysis.

In the plot, all trees with a diameter at breast height ≥1 cm were tagged, measured, mapped, and identified to species (40). The plant stand density, plant richness, plant diversity, and plant basal area were measured and calculated as environmental factors of woody plants. Plant richness refers to the number of species. Plant stand density indicates the number of individual trees. Plant diversity was calculated by reference formula (41). Plant basal area was calculated as π × *R*^2^, where *R* is the radius at a height of 1.3 m (42).

For each 20 × 20 m subplot, the elevation, convexity, aspect, and slope were measured using the methods described by Harms et al. (43) and Valencia et al. (44).

Hemispherical photographs were obtained using a Canon EOS 60D camera (Japan) at four corners of 20 × 20 quadrats at 1.3 m above the ground (45). Photographs were taken during either early dawn, late dusk, and overcast weather whenever possible to ensure the accuracy of the data (46). Three replicate photos were taken, and the photos showing the highest contrast between the sky and foliage were selected as the valid photo. The selected effective photographs were processed using the Hemi View woodland canopy digital analysis system. The average leaf angle, canopy cover (CC), total radiation, scattered radiation (SR), direct radiation, transmittance of light (LT), and leaf area index were obtained (47).

Spatial factors were derived from the principal coordinate analysis (PCoA) of a truncated distance matrix (PCNM). Based on quadrat coordinates, the geographical distances were converted to geospatial factors (48, 49). Data were log-transformed prior to statistical analysis when necessary. A forward selection procedure was used to select the PCNM variables by using the “pcnm” function in the vegan package (48).

### DNA extraction, PCR, and Illumina sequencing

The total soil bacterial DNA was extracted from 0.5 g of fresh soil samples by using the Fast DNA SPIN extraction kit (Mobio Laboratories, Carlsbad, CA, USA) according to the manufacturer’s instructions (32). The purified DNA concentration was determined using a spectrophotometer (Thermo Scientific, Wilmington), and its integrity was detected by 1% agarose gel electrophoresis. The V4-V5 region of prokaryotic 16S rRNA genes was amplified using the universal primer pair of 515F (5′-GTG YCA GCM GCC GCG GTA-3′) and 907R (5′-CCG YCA ATT YMT TTR AGT TT-3′) (50, 51). The PCR amplification cycles for 16S rRNA genes consisted of an initial denaturation at 95°C for 3 min, followed by 30 cycles of 95°C for 30 s, 55°C for 30 s, and 72°C for 45 s, and a final extension at 72°C for 10 min (52). The PCR product was purified and quantified as described previously (32). All libraries were sequenced on the Illumina HiSeq platform (Illumina Inc., San Diego, CA, USA) by using a paired-end (2 × 150 bp) approach. The sequencing and bioinformatics analyses were performed by Huada Gene Technology Co., Ltd., Shenzhen, China.

### Bioinformatics analysis

Raw paired-end FASTQ sequences were assembled using FLASH (v.1.2.11) under default settings (53). The obtained raw sequence data were analyzed and processed using the Quantitative Insights into Microbial Ecology pipeline against the compiled files; the procedures were described in detail by Yao et al. (54). A total of 7,329,751 sequences were obtained from all bacterial samples. After quality filtering, denoising, and chimera removal, the UCLUST algorithm was used to divide the sequences into different operational taxonomic units (OTUs) according to 97% similarity (55). Species annotation was carried out using the Greengenes database (http://greengenes.lbl.gov/).

### Analysis of the habitat generalists, specialists, and neutral taxa

The niche breadth was calculated as described by Pandit et al. (56) by using the Levins niche breadth index:


$$B_{j}=\frac{1}{\sum_{i=1}^{N}P_{ij}^{2}}$$

*B*_*j*_ represents the niche breadth of OTU *j* in the communities, while *P*_ij_ represents the relative abundance of OTU *j* in a given habitat *i* (i.e., each of the 120 samples was considered a “habitat”) (56, 57). A given OTU with a higher B value indicates a wider niche breadth. OTUs with wider niche breadth are more evenly distributed and more metabolically flexible than those with narrower niche breadth (58). The analysis was based on the function “Niche Breadth” in the R package “Spaa” (59).

Microbial communities were divided into generalists or specialists and neutral taxa based on the Levins niche breadth (1). The occurrences of OTUs generated by simulating 1,000 permutations (quasiswap permutation algorithms) were calculated using the EcolUtils R package. The OTUs were further classified as generalists, neutral taxa, and specialists based on their occurrence and by using permutation algorithms as implemented in EcolUtils. Generalists have wider fundamental niches than specialists (60). In the present study, an OTU was considered a generalist or specialist based on whether the observed occurrence exceeded the upper 95% confidence interval or fell below the lower 95% confidence interval, and the OTUs were considered neutral taxa if the observed niche breadth was within the 95% confidence interval range (61). In total, 5.97% of OTUs were classified as generalists, 41.14% as specialists, and the remaining 52.89% as neutral taxa.

### Statistical analyses

The richness indices of all samples were calculated using the diversity function in the “Vegan” package (62). The Kruskal-Wallis method was used to test for differences in the bacteria richness and niche breadth in the four communities (63, 64).

Beta-nearest taxon index (beta NTI) and Raup-Crick metric (RC-Bray) values were used in null model analyses to assess the influence of different ecological processes, both stochastic and deterministic, on bacterial community assembly (65). When |βNTI| > 2, deterministic processes govern the observed community turnover between pairs of communities, whereas |βNTI| < 2 suggests that stochastic processes drive community succession (28). Meanwhile, the neutral community model was employed to estimate the potential contribution of neutral processes to community assembly (66). A best-fit distribution curve between OTU occurrence frequency and its relative abundance was generated using nonlinear least-squares analysis (67). In this model, a single free parameter *m* is used to describe the migration rate. A higher *m* value indicates that microbial communities are less influenced by environmental constraints (68). The *R*² value represents the goodness-of-fit to the model and was calculated according to the “Östman method” (69). When *R*² approaches 1, it suggests that the community assembly is fully consistent with stochastic processes. Model computations were performed using R version 3.6.1.

Furthermore, the effects of deterministic processes on the bacterial community assembly were tested by checking the deviation degree of each observation index from the average value of the null model (C-score) (70). The calculation method of standardized effect size (SES) was based on the research method of Gotelli et al. (71). The magnitude of SES is interpreted as the strength of the effect of deterministic processes on the assemblage, where the higher the absolute value of SES, the stronger the relative contribution of deterministic processes (72). C-score was determined using the sequential swap randomization algorithm with the package “EcoSimR” in R version 3.6.1 (73).

A network analysis method was used to reveal the co-occurrence patterns of generalists, neutral taxa, and specialists in the study area. Network analysis data were visualized using Gephi software (74). The topology structure of the bacterial network was evaluated based on the modularity index. Each node indicates a given OTU, and each edge represents a significant correlation between two OTUs. Degree represents the number of edges connecting each node to the rest of the nodes in the network. For the bacterial community structure, the R language “igraph” package is used to build and analyze the network (75).

In the present study, the partial least-squares path modeling (PLS-PM) was used to quantitatively analyze the direct and indirect effects of light, topography, PCNM, and woody plant factors on bacteria richness. The methods of Chu et al. (76) and Wang et al. (77) were used to analyze the direction and intensity of the effect of environmental variables on species richness. In PLS-PM, each latent variable includes one or more indicator variables. For example, the latent variable (PCNM) includes two indicator variables (PC2 and PC3); plant includes BA (plant richness) and DEN (plant stand density), light includes CC, SR, and LT, and topography includes ASP (aspect). The relationships among these block variables were quantified with path coefficients. The goodness-of-fit index was used to estimate the prediction performance of models (78). PLS-PM was performed using the package “plspm” in R 4.0.1 (41).

## RESULTS

### Diversity and niche breadth of bacterial communities

**Fig 2:**
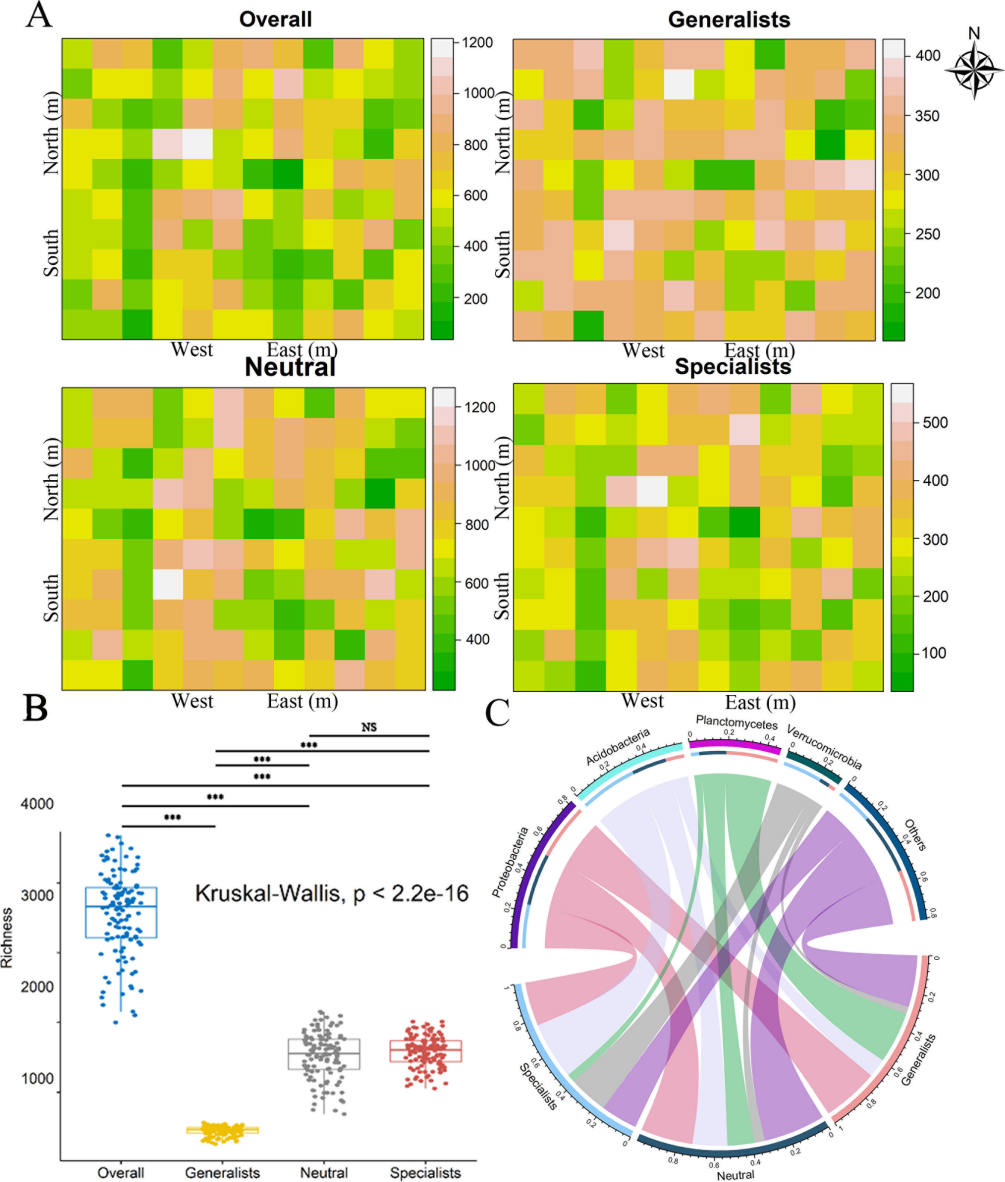
Spatial distribution and species composition of overall taxa, specialists, generalists, and neutral taxa in Baiyun National Forest Park. (**A**) Distribution map of bacterial OTU species in 120 sample plots. (**B**) Richness index of bacterial communities. (**C**) Species composition of bacterial communities.

Habitat generalists, specialists, neutral taxa, and all bacterial communities had similar spatial distribution patterns (Fig. 2A). In comparison with the specialists, the spatial distribution of the generalists is more extensive. The Kruskal-Wallis test showed significant differences in species richness among overall species, generalists, neutral taxa, and specialists (Fig. 2B, *P* < 0.001). More generalists were identified within Proteobacteria and Planctomycetes, while more specialists were associated with Acidobacteria and Verrucomicrobia (Fig. 2C).

### Relative importance of deterministic and stochastic processes

**Fig 3:**
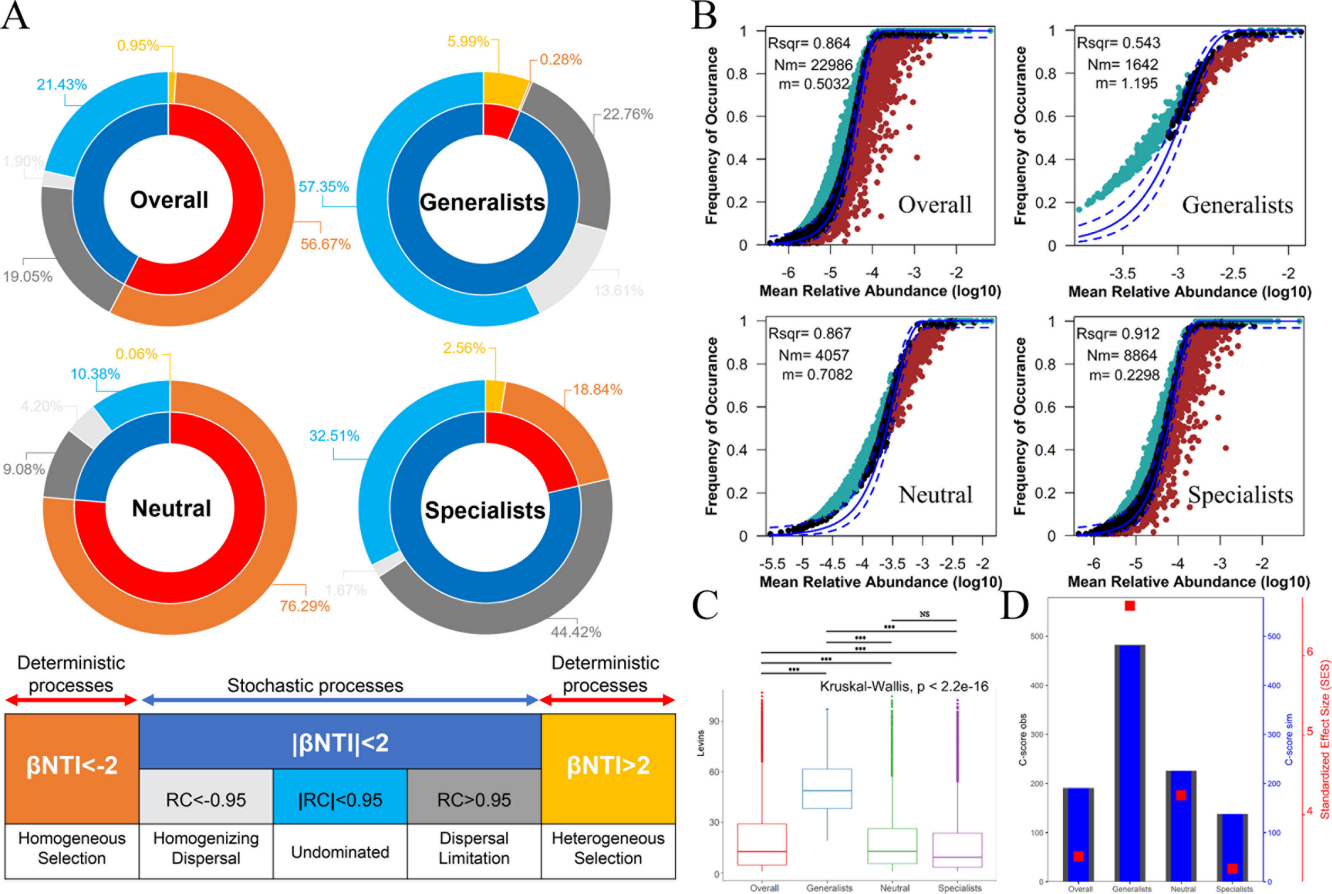
Ecological processes of the bacterial communities in Baiyun Mountain National Forest Park. (**A**) Assessment of the influence of stochastic and deterministic processes on soil bacterial community assembly based on a null model. The inner circle represents the contribution of stochastic and deterministic processes to community construction. The outer ring represents the detailed ecological processes assigned to stochastic and deterministic processes. (**B**) Neutral model applied to assess the effects of random dispersal on the soil bacteria. Rsqr indicates the goodness-of-fit to the neutral model. Nm indicates the metacommunity size times immigration. m indicates the estimated migration rate. The solid blue lines indicate the best fit to the neutral model, and dashed blue lines represent 95% confidence intervals around the model prediction. (**C**) Comparison of the mean niche breadth of four bacterial taxa. The Kruskal-Wallis test at *P* < 0.05. (**D**) C-score metric based on null models. The values of observed C-score (C-scoreobs) > simulated C-score (C-scoresim) indicate non-random co-occurrence patterns. Standardized effect sizes <−0 and >0 represent aggregation and segregation, respectively.

The results of the null model analysis indicated that habitat generalists, specialists, neutral taxa, and the overall bacterial community were all influenced by a combination of deterministic and stochastic processes (Fig. 3A). Both generalists (stochastic processes: 93.7%; deterministic processes: 6.3%) and specialists (stochastic processes: 78.6%; deterministic processes: 21.4%) were predominantly governed by stochastic processes. The relationship between the distribution and relative abundances of bacterial taxa was well-described by the neutral community model (Fig. 3B). The neutral community model well-fitted the frequency of microbial OTU (86.4%) and played an important role in bacterial community assembly. Generalists, neutral taxa, and specialists explained 54.3%, 86.7%, and 91.2% of the community variance, respectively. The relatively higher *m* value for generalists than for specialists (1.195 vs 0.2298) suggests that generalists are highly diffuse and are less restricted by the environment. In addition, compared with the specialists, the generalists showed a wider niche breadth (Fig. 3C). More importantly, the C-score showed that SES decreased with changes in specialists (Fig. 3D), neutral species, and generalists, suggesting the decreased importance of deterministic processes for bacterial subcommunities assemblage. Both habitat generalists and specialists were more strongly driven by stochastic rather than deterministic processes.

### Co-occurrence networks of bacterial microbial communities

**Fig 4:**
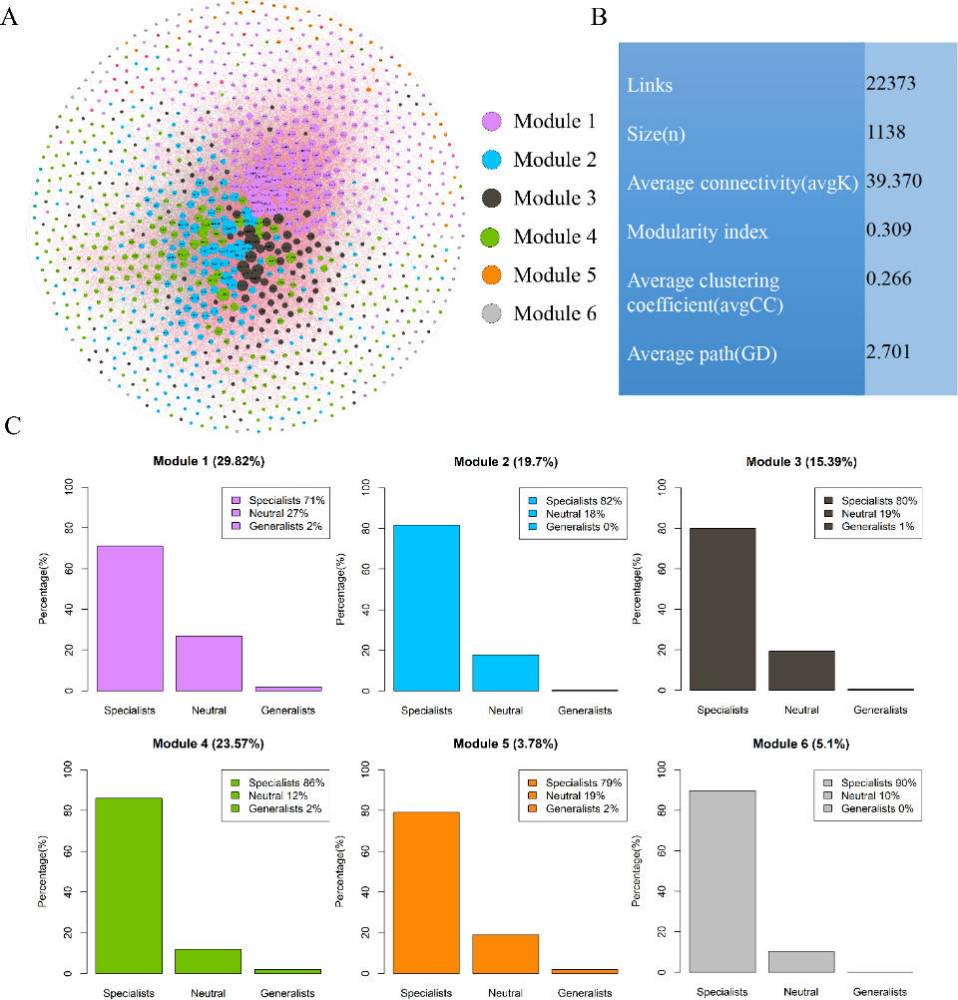
Co-occurring network colored by modularity class for soil bacteria in Baiyun Mountain National Nature Reserve. (**A**) The co-occurrence patterns among OTUs revealed by network analysis. The red lines show positive correlations between nodes, and the green lines show negative relationships. Each node represents different OTUs, and the colors of the nodes indicate different modules. Modules 1–6 display different colors. A group of OTUs in one module means that these OTUs have more interactions among themselves and fewer associations with other modules. (**B**) Topological properties of the co-occurrence network of soil bacterial communities. (**C**) Relative abundance of specialists, neutral species, and generalists (OTUs) in the main modules.

The bacterial community network was clearly divided into four major modules, accounting for 88.48% (module 1–module 4) of the whole network (Fig. 4A through C). The co-occurrence network showed high ratios of positive correlations and consisted of 1,138 nodes (OTUs) and 22,373 edges (average connectivity, 39.370). Among all nodes, 15 and 914 OTUs belonged to generalists and specialists, respectively. The average path length was 2.701 edges, and the clustering coefficient and modularity index were 0.266 and 0.309, respectively. Bacterial communities were dominated by taxa preferring specialists in all modules, but only a few generalists were present. In the co-occurrence network, five phyla (Proteobacteria, Acidobacteria, Planctomycetes, Verrucomicrobia, and Chloroflexi) were widely distributed, accounting for 73.8% of all nodes (Fig. S1).

### Direct and indirect effects of environments on bacterial community

**Fig 5:**
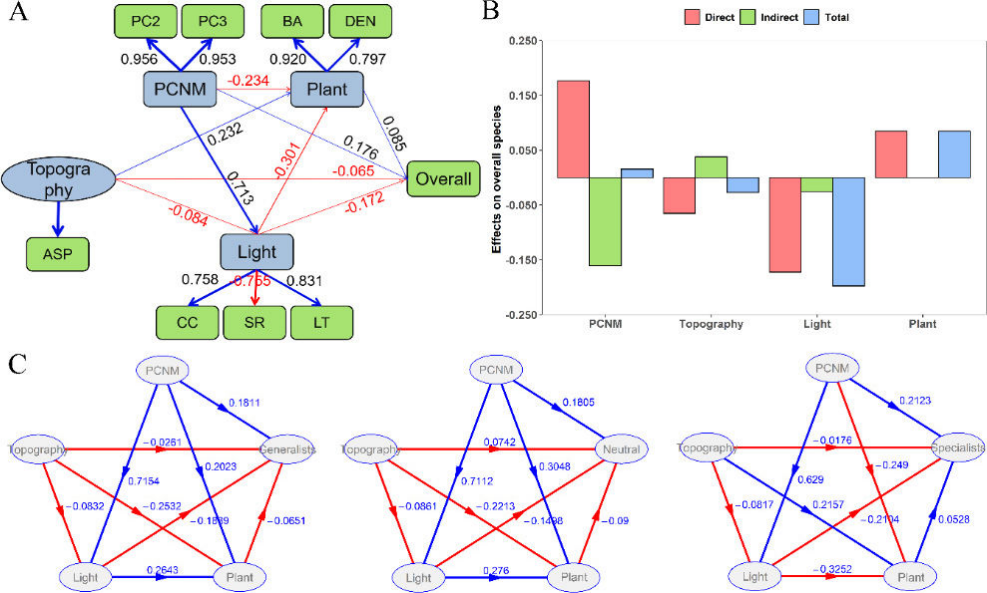
PLS-PM showing the direct and indirect effects of different factors on bacteria richness. (**A**) PLS‐PM was used to examine the linkages among light, PCNM, plant, topography, and overall species richness. The blue line indicates positive correlation, while the red line indicates negative correlation. The arrow color and width indicate the strength of the relationship. Numbers on the lines out of the PLS-PM were the “weight” contributions. (**B**) The positive and negative effects of PCNM, topography, light, and vegetation on bacterial richness. (**C**) Direct and indirect effects of environmental factors on generalists, neutral species, and specialist richness.

For bacteria richness (Fig. 5 through B), PCNM (spatial effect) had the highest path coefficient of 0.176, which can be attributed to the strong spatial structuring of the plot environment variation. The goodness-of-fit of total bacterial richness and environmental factors was 0.496, which is higher than 0.35, indicating that the model was reliable. The effects of light and PCNM on bacterial richness were statistically significant; the direct and indirect contribution rates of PCNM to bacterial richness were 17.61% and 16.07%, respectively, and the direct and total contribution rates of light to bacterial richness were 17.21% and 19.76%, respectively.

PCNM and light had similar positive or negative effects on the richness of specialists, generalists, and neutral taxa. The path coefficients of light (21.04%) and PCNM eigenvectors (21.23%) of the specialist richness were higher than those of generalists (light, 18.89%; PCNM eigenvectors, 18.11%), indicating that light and PCNM had a greater effect on specialists than generalists.

## DISCUSSION

### Assembly of bacterial communities

Our results clearly demonstrate the important roles of both stochastic and deterministic processes in bacterial community assembly (Fig. 3). Based on the null model, the neutral community model, niche breadth, and C-score analyses, we found that both generalist and specialist taxa in forest ecosystems were more influenced by stochastic processes than by deterministic ones. Habitat generalists showed a higher degree of stochasticity compared to specialists. Similarly, Zou et al. found that both generalist and specialist planktonic bacterial taxa in lakes and reservoirs were mainly governed by stochastic processes (79). In our study, habitat generalists had broader niche breadths, which allowed them to tolerate a wider range of environmental conditions (80). This may explain why they were more strongly affected by stochasticity. Meanwhile, the C-score results indicate that as the niche breadth decreases from broad (habitat generalists) to narrow (habitat specialists), the SES value declines, indicating a reduced influence of deterministic processes and an increased dominance of stochastic processes. This reflects the differences in ecological strategies among different functional groups in terms of spatial dispersal and environmental adaptation, further supporting that dispersal limitation is the dominant process shaping specialist communities.

However, habitat specialists in farmland microbial communities often exhibit strong preferences for specific environmental conditions, rendering them more susceptible to species sorting, a form of deterministic process (81). This is inconsistent with our findings. In our study, dispersal limitation contributed the most to the assembly of specialist communities, with a maximum contribution of 44.42%. This could be due to their narrower spatial distributions (Fig. 2A), narrower niche breadths (Fig. 3C), and lower dispersal abilities. These traits may prevent them from crossing spatial barriers to reach suitable habitats. Specialists may try to escape unfavorable environments, but due to their weak dispersal capacity, they are more likely to be limited by dispersal (82). In environments with low human disturbance, stochastic processes tend to dominate (83). Our study site is a national nature reserve that has remained nearly undisturbed for over a century. The low environmental filtering pressure in such a stable environment may have reduced deterministic constraints on specialists, thereby increasing their susceptibility to stochastic processes (81). In summary, in temperate deciduous broadleaf forests, both specialists and generalists are more strongly shaped by stochastic processes. Generalists, however, are influenced by stochasticity to a greater extent than specialists.

### Coexistence patterns of the habitat generalists and specialists

In this study, habitat specialists contributed more to the stability of the entire bacterial network than generalists. All six densely connected modules were dominated by habitat specialists (typically accounting for over 70%), while widely distributed generalists were extremely rare (Fig. 4). This specialist-dominated modular structure suggests a significant degree of niche differentiation within the community. Within the co-occurrence network, habitat specialists were more likely to function as intra-module hubs, playing a pivotal role in maintaining the structural integrity and functional coherence of individual modules. In contrast, generalists, despite having fewer intra-modular associations, tended to act as connectors across modules, thereby contributing to the overall functional redundancy and resilience of the microbial community (81). Our analysis showed that habitat specialists comprised 80.32% of the network nodes, while generalists accounted for only 1.32%, highlighting the critical structural role of specialists. This is consistent with previous findings (11), showing that specialists tend to form more complex and stable network structures (6, 28).

Within these modular structures, keystone taxa were frequently located at the module cores, characterized by a high degree and high betweenness centrality, linking multiple functional pathways and enhancing module stability (84). Our study further found that all keystone taxa belonged to habitat specialists, emphasizing their irreplaceable role in maintaining module functions. The loss of these central nodes could lead to a rapid collapse of intra-module cooperative relationships and result in disruptions to ecological processes and functional losses (85). Therefore, specialists not only dominate niche partitioning but also play a crucial role in sustaining both network integrity and ecosystem functioning.

### Direct and indirect effects of environmental factors on bacterial community structure

In the present study, the variation in bacterial richness is mainly explained by the light and space eigenvectors in temperate deciduous broad-leaved forests (Fig. 5). Light had a direct negative effect on bacterial richness, which is consistent with previous studies (86). Under the dense canopy, the light is weak, the water evaporates less, and the soil moisture is very high (74). In addition, humus is very abundant in low-light conditions, which is beneficial to the reproduction and survival of soil bacteria (87). Therefore, soil bacteria may have a strong distribution preference for low-light habitats. Spatial eigenvectors can also affect bacterial richness directly and indirectly, which is consistent with other studies (87). The 17.6% variability explained by spatial eigenvectors may reflect dispersal and biological interactions (88, 89). The explanatory power of topography and woody plants to bacterial richness is weak, which is consistent with a previous report (87), possibly because of the limited topographic variability and the aggregation distribution of species. This condition reduced the community heterogeneity.

In the present study, bacterial habitat specialists were more likely to be affected by light and space eigenvectors than generalists, which was consistent with previous findings (35). Habitat specialists had a higher degree of response to environmental change than generalists, because habitat generalists showed broad environmental tolerance, while habitat specialists exhibited a narrow range of environmental tolerances, being more sensitive to and less resistant to environmental changes (35). Light and space eigenvectors are important drivers of habitat specialists, which might influence the richness of habitat specialists in part by limiting bacterial dispersal. This finding suggests that habitat specialists may have stringent requirements for environmental conditions, and their living conditions largely depend on these specific or combined environmental factors (15). Habitat specialists face extinction if severe environmental disturbances occur. A relatively large proportion of species variation in our data cannot be explained by light and spatial data, partly because of random dispersal, but it might also include deterministic changes caused by unmeasured environmental variables (soil physical and chemical properties, etc.) (90, 91).

### Conclusion

**Fig 6:**
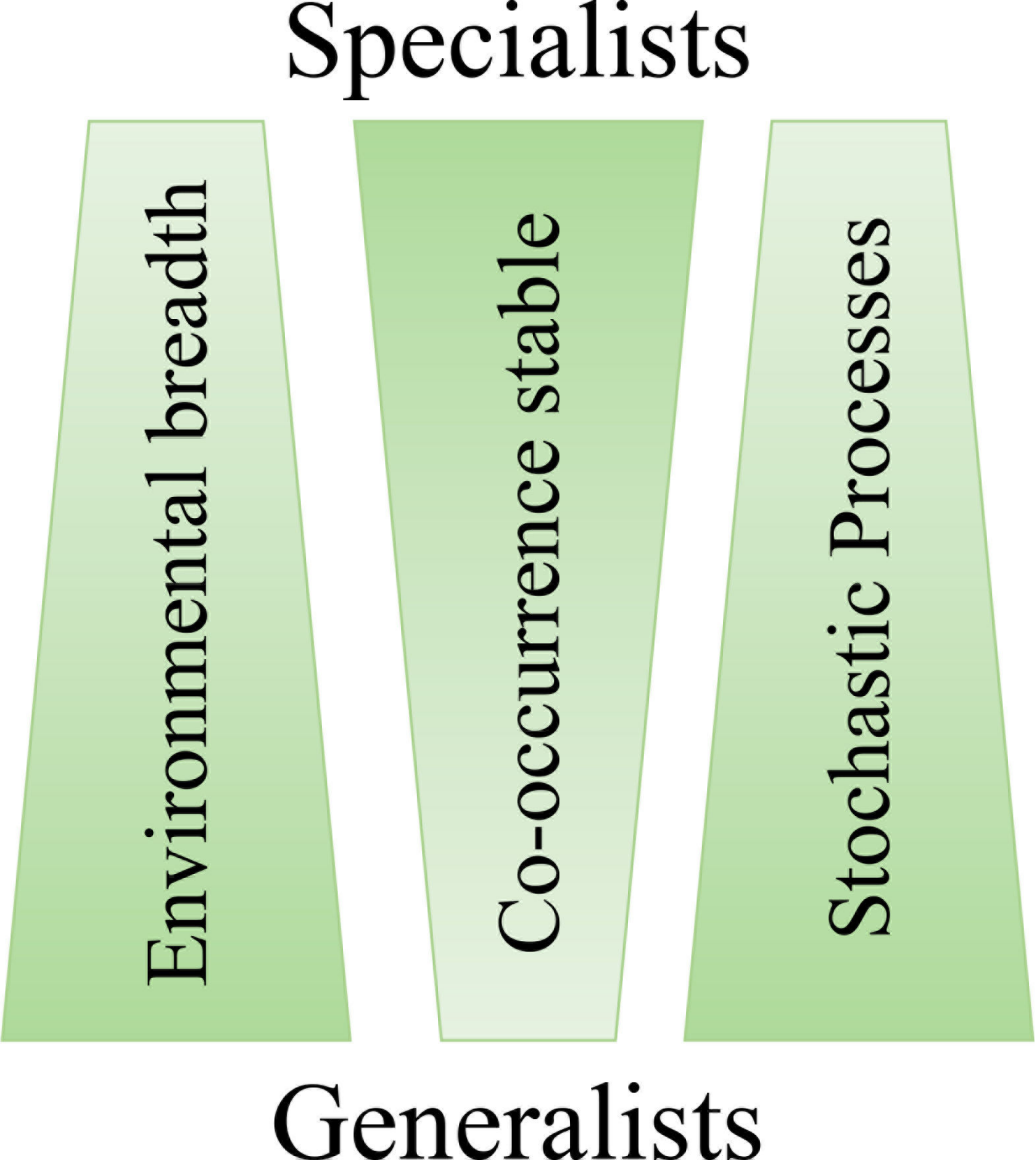
Conceptual map showing the environmental breadth, co-occurrence pattern, and stochastic processes in the assembly of soil bacterial habitat generalists and specialists in the mountain forest ecosystem.

A conceptual framework was designed to describe the community assembly process of soil bacterial habitat generalists and specialists in temperate deciduous broad-leaved forests, the environmental breadth, and the role in co-occurrence network stability (Fig. 6). Habitat generalists exhibited broader environmental breadths than specialists, and their community assembly was predominantly influenced by stochastic processes in temperate deciduous broad-leaved forests. In contrast, habitat specialists contributed more significantly to the stability of the entire co-occurrence network than generalists. Overall, our findings may have important implications for the formation and maintenance of soil bacterial diversity in temperate deciduous broad-leaved forests and help in predicting the response of bacterial communities to surrounding environmental changes.

## Data Availability

All raw read data of 16S genes have been submitted to the NCBI GEO under the accession number PRJNA633088.

## SUPPLEMENTAL MATERIAL

Figure S1 aem.00992-25-s0001.docx Relative abundance at the level of phyla.

ASM does not own the copyrights to Supplemental Material that may be linked to, or accessed through, an article. The authors have granted ASM a non-exclusive, world-wide license to publish the Supplemental Material files. Please contact the corresponding author directly for reuse.
