# Supplementary figures and images for: Distinct strategies of soil bacterial generalists and specialists in temperate deciduous broad-leaved forests
Source: Appl Environ Microbiol. 2025 Jul 30;91(8):e00992-25. doi: 10.1128/aem.00992-25 (PMC12366338; doi:10.1128/aem.00992-25)

Supplementary material


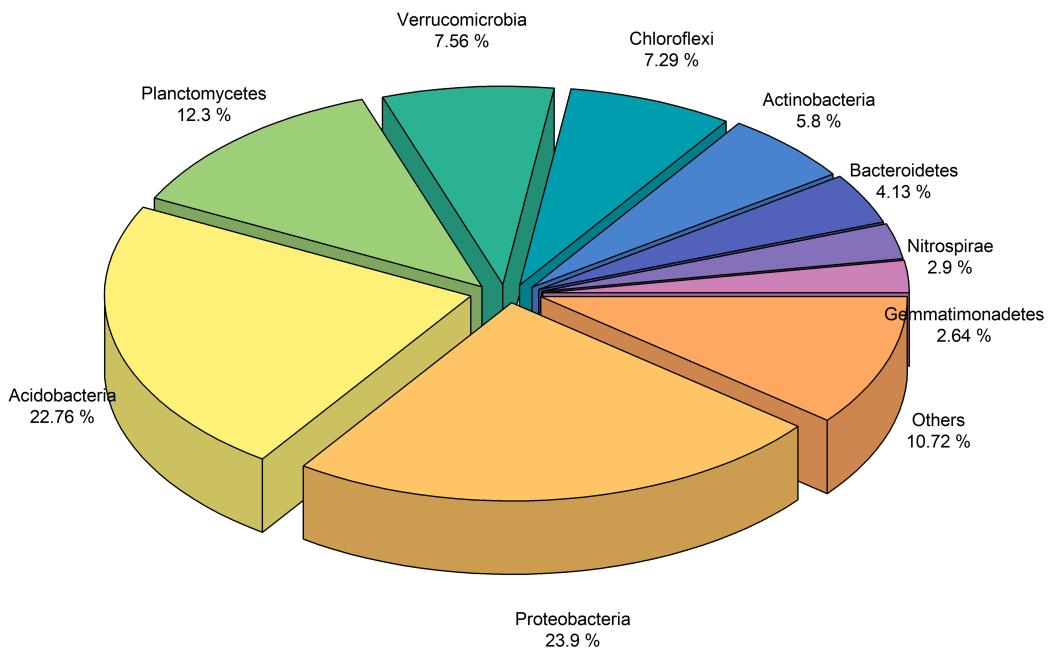


S.Fig. 1 The relative abundance at the level of phyla

Supplement: Figure S1 — Relative abundance at the level of phyla. [file aem.00992-25-s0001.docx]
